# American Microscopes

**Published:** 1848-01

**Authors:** 


*American Microscopes.*—A good Microscope has become so essential to the physician, that no apology is needed for calling the attention of our readers to this subject. We scarcely know whether anatomy, physiology, or pathology is most indebted to its revelations; but we are certain that no one who wishes to push his researches into the very *penetralia* of the human body, will rest satisfied without being in possession of such an instrument. To it we are indebted for much of our knowledge of what takes place in the organism, as the capillary circulation, and the early processes of development both in plants and animals; to say nothing of the nature of those changes, which are constantly taking place as the result of abnormal rction.

It gives us much pleasure to state, that we are at length enabled to procure a better microscope at home, than we are abroad, and at a much more reasonable price. We learned some time since, that Mr. Charles A. Spencer, of Canestota, N. Y., was manufacturing microscopes, telescopes, etc., equal in all respects to those imported; but it is not until very recently that we have been put in possession of facts and testimony which establish beyond dispute their superiority to all others. Professor Baily, of West Point, in a recent letter to Professor Torrey, (dated Sept. 27th, 1847,) thus speaks of one of Mr. Spencer's instruments.

"I have just had very great pleasure in an interview with Mr. Spencer, and in looking through his microscopes, I am proud as an American of their performance. You know we tried in vain with our highest powers to see the cross lines on the *Navicula Hippocampus*. Mr. Spencer's lenses show them beautifully, and they may even be seen by his middle power. He has shown me lines on several objects on which I could never before find any trace of them. I feel therefore no hesitation in pronouncing his microscopes *far superior tc Chevalier's*, and they show all the lines in the most difficult objects, which I could see with the Lowell instrument, at Boston. This is a triumph for American art, in which we should all rejoice, and feel it a duty and pleasure to spread the knowledge of."

Mr. Spencer has been lately engaged to furnish as good a microscope as he can make, of the large Chevalier form, for the Smithsonian Institution, without regard to cost; also, one for Princeton and Geneva Colleges. The large Chevalier form, which costs, with the duties, and without polarizing apparatus, etc., $250, is made by Mr, Spencer, with a full range of powers, for $200; and with polarizing apparatus, micrometers, compressor, eclectric conductor, camera lucida, etc., for $225. The small Chevalier, which costs with these powers and no auxiliary apparatus, $100, is furnished by Mr. S. for the same price; and the same price; and the same with a greater range of powers and apparatus, for $125 to $150. His verticle and oblique instruments, vary in price from $75 to $150, with lenses far superior to any of the Chevalier that we have examined. We confidently recommend Mr. Spencer's microscopes to all who wish to supply themselves with such an instrument, believing that their highest expectations will not be disappointed.—*New-York Journal of Medicine and Collateral Sciences.*

The Medical Faculty of Buffalo Medical College have also ordered from Mr. Spencer a microscope of the large Chevalier form, with a full range of powers, to be ready for use before the opening of the spring term of lectures.

